# Blood Counting in General Practice

**Published:** 1902-05-31

**Authors:** 


### BLOOD COUNTING IN GENERAL PRACTICE.

A GOOD deal is being heard at present about the counting of leucocytes, and the importance of this as a means of diagnosis in certain conditions is being much insisted on, more especially of course by those who are connected with large hospitals and have at their disposal the services of skilful clinical assistants. As qualifying to some extent the general utility of the proceeding it is worth bearing in mind that in the opinion of some, blood counting is work which can only be properly undertaken by the specialist. Dr. Warren Simmons [1] has lately laid it down that the technique of a blood examination is distinctly in the realm of the specialist in pathology, and that to him should be entrusted the responsibility of this important procedure. Such an examination should, he says, be conducted by men who possess the necessary knowledge, exact methods, special laboratory facilities, and above all a skill in their work that only comes from an extensive experience, and he adds that the blood examination as conducted by the average practitioner will be of no value whatever. In all this Dr. Simmons obtained considerable support from several physicians who discussed his paper, one of whom said that no leucocyte count, or for the matter of that many other of the microscopic tests now so often employed, can be relied upon unless done by a man who is doing it all the time. We record these views because they may perchance give support to some of our profession who, not having kept up their familiarity with clinical laboratory work, would not feel themselves competent for such investigations. At the same time we must express our dissent from the general proposition that useful investigations of this nature can only be properly undertaken by those who are especially and exclusively engaged in laboratory work. It must be remembered that even in clinical laboratories the actual work is done by young men who could not by any stretch of language be properly described as pathological specialists, and we feel assured that if medical men would but keep up by frequent practice with the microscope and the test tube, the manual skill which they acquired in their "clerking" days, they might safely and with much advantage to their patients undertake a great deal of that sort of work which, partly from laziness and partly from a dislike to undertake responsibility, is being allowed to drift into other hands. That a blood count takes up a lot of time we admit, but that none but the elect can do it we do not believe for a moment.

[1] Brooklyn Med. Jour., May 1902.

